# In Silico Approach for SAR Analysis of the Predicted Model of DEPDC1B: A Novel Target for Oral Cancer

**DOI:** 10.1155/2016/3136024

**Published:** 2016-02-29

**Authors:** Palak Ahuja, Kailash Singh

**Affiliations:** ^1^Department of Biotechnology, Faculty of Sciences, Jamia Hamdard, New Delhi 62, India; ^2^School of Biological Sciences, The University of Hong Kong, Pok Fu Lam Road, Hong Kong

## Abstract

With the incidence rate of oral carcinogenesis increasing in the Southeast-Asian countries, due to increase in the consumption of tobacco and betel quid as well as infection from human papillomavirus, specifically type 16, it becomes crucial to predict the transition of premalignant lesion to cancerous tissue at an initial stage in order to control the process of oncogenesis. DEPDC1B, downregulated in the presence of E2 protein, was recently found to be overexpressed in oral cancer, which can possibly be explained by the disruption of the E2 open reading frame upon the integration of viral genome into the host genome. DEPDC1B mediates its effect by directly interacting with Rac1 protein, which is known to regulate important cell signaling pathways. Therefore, DEPDC1B can be a potential biomarker as well as a therapeutic target for diagnosing and curing the disease. However, the lack of 3D model of the structure makes the utilization of DEPDC1B as a therapeutic target difficult. The present study focuses on the prediction of a suitable 3D model of the protein as well as the analysis of protein-protein interaction between DEPDC1B and Rac1 protein using PatchDock web server along with the identification of allosteric or regulatory sites of DEPDC1B.

## 1. Introduction

The association of cervical carcinomas with human papillomaviruses (HPVs), a group of small DNA viruses, was first recognized by Meisels et al. [1, 2]. Apart from being associated with cervical carcinomas, human papillomaviruses, particularly type 16, were found to be one of the major causes of oral squamous cell carcinoma (OSCC), which is the sixth most common cancer worldwide [3–5].

At a molecular genetic level, the high risk HPV 16 positive oral squamous cell carcinomas are known to express the E6 and E7 gene products which are recognized for their oncogenic potential. The E6 gene product tends to either associate with p53, forming a stable complex to promote the proteolytic degradation of p53, or downregulate the activity of effective p53 via targeting CBP/p300 (transcriptional coactivator) and therefore interferes with its function and deregulates the cell cycle [6–8]. In addition, E7 protein binds to a tumor suppressor protein, pRb, resulting in the activation of E2F (transcription factor) which further stimulates the expression of proteins critical for DNA replication [9, 10]. Normally, this unprepared onset of S-phase results in the initiation of apoptosis via p53, which, however, will not be initiated in the case of HPV infected cells owing to p53 inactivation by the viral E6 protein [11]. The regulation of the E6 and E7 gene products is mediated by another viral protein, E2. E2 is known to interact with the two E2 DNA binding sites residing in close proximity with E6/E7 promoter region [12–14]. Thus, HPV E2 being a repressor of viral oncogenic protein is termed as viral tumor suppressor, which, however, upon the integration of HPV 16/18 genome into the host genome results in the disruption of E1 and E2 open reading frames (ORF) followed by unregulated transcription of the oncogenic proteins, E6 and E7. This upregulated transcription therefore results in the initiation of oral oncogenesis [15].

**Figure 1:**
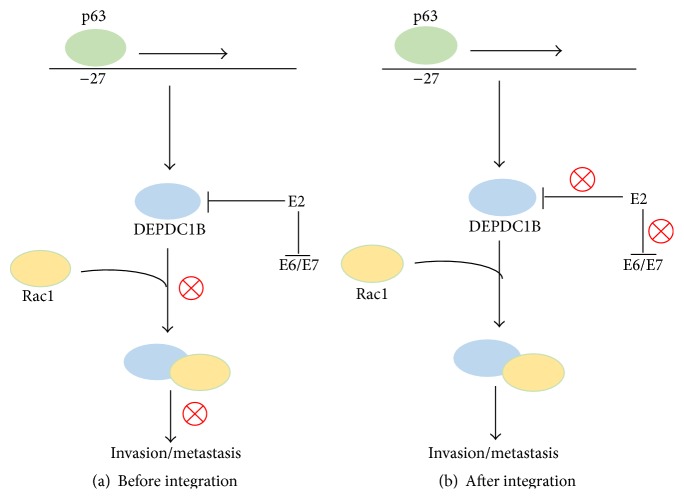
Regulatory mechanism involved in the action of DEPDC1B leading to invasion/metastasis, before (a) and after (b) the integration of viral genome into the host genome, resulting in the disruption of E2 ORF, followed by oral carcinogenesis.

E2 is known to interact with and downregulate several proteins (CPB2, HSPBAP1, RBM26, etc.), one of them being DEPDC1B, which recently was found to be overexpressed in oral cancer [16, 17]. This can be possibly explained by the disruption of the E2 ORF upon the integration of viral genome into the host genome. DEPDC1B protein contains two characteristic domains: DEP, which enables the protein to interact with the G protein coupled receptors as well as negatively charged membrane phospholipids, necessary for Wnt signaling [18, 19], and RhoGAP, responsible for Rho GTPase signaling [20]. The regulation of its expression is speculated to be positively controlled by p63, supported with the fact that a p63 binding site exists at −27 Kb from the transcription start site of DEPDC1B; however, the mechanism is still not clear (Figure 1) [21]. The precise function of DEPDC1B is uncharacterized; however, in recent studies it has been shown to promote cell growth, invasion, and anchorage-independent growth of oral cancer cells, the function being mediated by the direct physical interaction of DEPDC1B with Rac1 protein [17]. Structurally, Rac1 consists of 6 *β* strands (5 parallel and 1 antiparallel) and 8 helices (six *α* helices and two 3^10^ helices) and it is known for its function in regulating the machinery controlling assembly as well as disassembly of cytoskeletal elements, cell cycle regulation, contact inhibition, cellular growth, and proliferation mediated via activation of NFkB [22–27]. With all these processes mediated by Rac1, it emerges as one of the regulators of oncogenesis [28], which itself is positively regulated by DEPDC1B [17], making it a potential protein based biomarker and a therapeutic target.

However, to have a better understanding of DEPDC1B meditated oral carcinogenesis and its role as a therapeutic target and a potential biomarker for diagnostic and prognostic purposes, it is important to understand the structural and functional aspects of the protein. The study, therefore, focuses on designing a 3-dimensional model of the structure of the protein under study as well as investigating the interacting amino acids between DEPDC1B and Rac1 in order to understand the biochemical aspect of it.

## 2. Materials and Methods

### 2.1. Retrieval of the Primary Sequence of DEPDC1B

The primary sequence of the protein, DEPDC1B (529 amino acid residues and 61.77 Kda in size) (UniProt ID: Q8WUY9), was obtained from UniProt (http://www.uniprot.org/) [38] in FASTA format.

### 2.2. Template Selection of DEPDC1B and Homology Modeling

**Figure 2:**
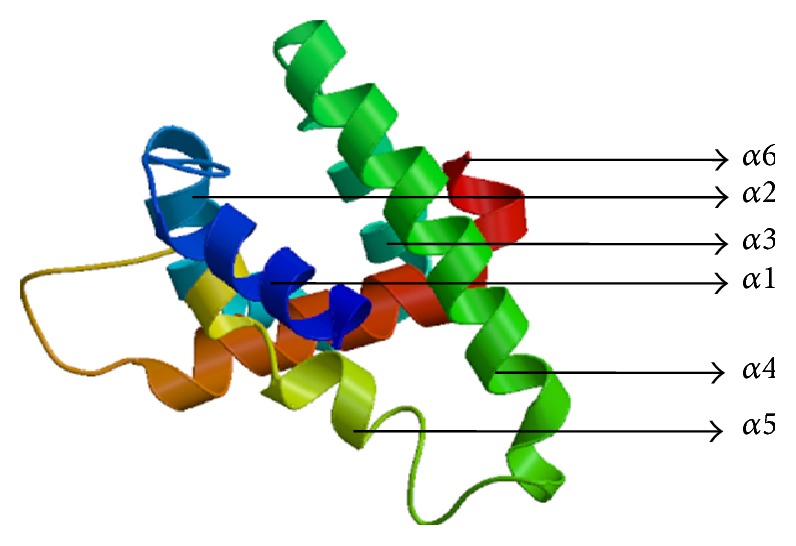
3D structure of the predicted model of DEPDC1B.

**Figure 3:**
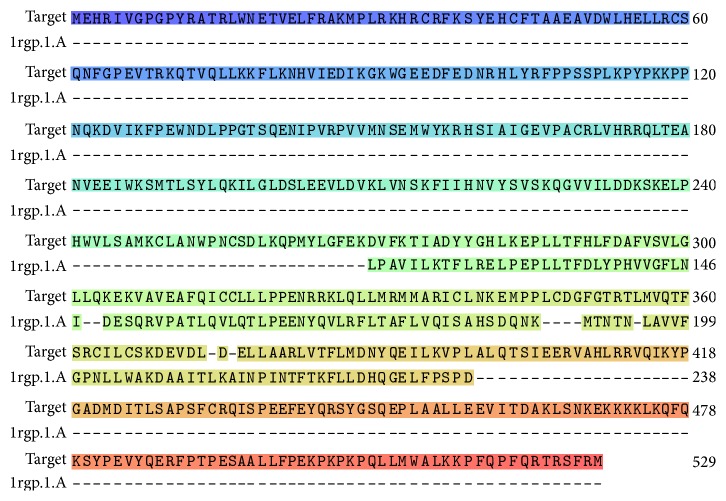
Sequence alignment of the template (PDB ID: 1RGP) and the target protein, DEPDC1B using Swiss model [29–32].

**Figure 4:**
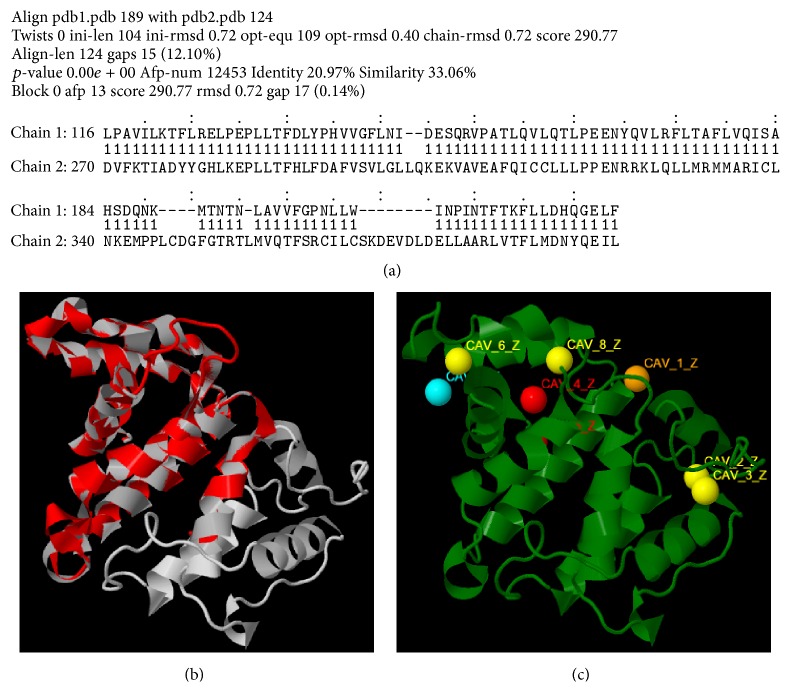
Depiction of (a) pairwise structural alignment between the template (chain 1) and modeled target protein, DEPDC1B (chain 2); (b) superimposition of template (grey) upon the target protein (red), using FATCAT [33]; and (c) active site prediction of template using PARS [34].

Multiple 3D models of the protein were developed and its energy minimized with the help of Swiss model (http://swissmodel.expasy.org/) [29–32], where the best model (Figure 2) was selected out of all, followed by its sequence alignment (Figure 3) which showed a sequence similarity of 23.97% with the template, that is, the GTPase-activation domain of RhoGAP (*Homo sapiens*), sequestered from RCSB PDB (PDB ID: 1RGP) [39, 40]. Additionally, pairwise structural alignment between the template and the modeled target protein was performed in order to predict structural similarity between the two, using the server FATCAT (Figures 4(a) and 4(b)) [33].

### 2.3. Visualization and Model Validation

**Figure 5:**
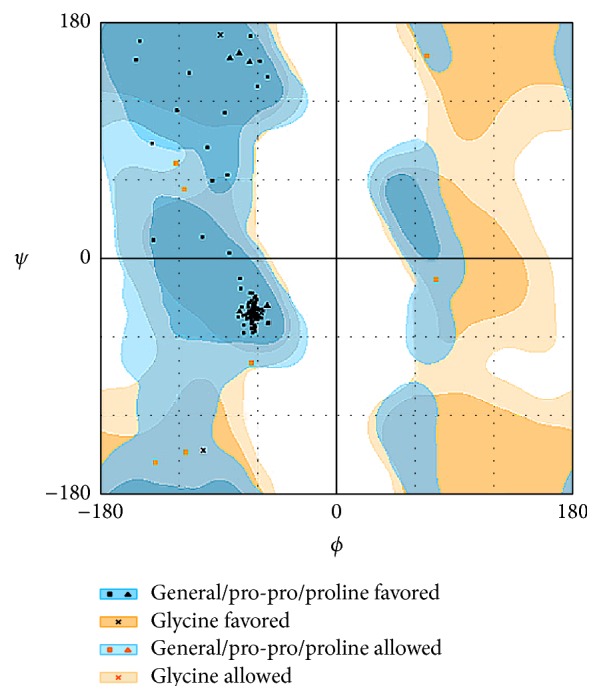
Ramachandran plot generated using RAMPAGE, indicating the amino acid residues in the favorable region [35].

The structure of the protein was predicted by Swiss modeling and visualized using PyMol version 1.3 [41], followed by the validation of the 3-dimensional structure using SAVES (http://services.mbi.ucla.edu/SAVES/), where the stereochemical quality of the 3D model obtained was verified using a program PROCHECK [42, 43] for the purpose of selecting the best model. VERIFY 3D structure evaluation server [44, 45] was used for 3D-profiling of the residues, ERRAT [35] for the verification of protein structures for evaluating the crystallographic model building and refinement progress, and RAMPAGE for the generation of Ramachandran plot to investigate the quality of protein structure (Figure 5) [46]. The *Z*-score was obtained by submitting the model to ProSA [47].

### 2.4. Retrieval of 3D Model of the Rac1

The structure file of the protein (PDB ID: 1I4D) [48] was retrieved by the RCSB PDB in the PDB format and was then used for studying the protein-protein interaction between the two proteins.

### 2.5. Docking Study

**Figure 6:**
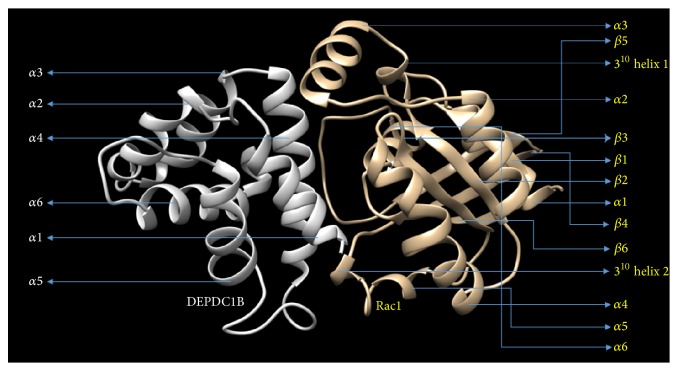
Docking interaction between DEPDC1B (light grey) and Rac1 (yellowish golden) visualized by chimera [36] and docking done using PatchDock server.

Docking of DEPDC1B with the Rac1 protein was conducted using the web server PatchDock/firedock (http://bioinfo3d.cs.tau.ac.il/PatchDock) (Figure 6) [49, 50], an online tool for protein docking designed for the purpose of the identification of the interaction sites between the two, wherein the molecular surface of the protein is divided into patches as per the molecular shape followed by the comparison between the patches in order to produce a group of transformations. These transformations are then ranked as per the geometric complementarity score and each transformation is assigned with a PatchDock score as well as atomic contact energy. Finally, the atomic dissolution energy of the derive complex is estimated and the redundant solution was rejected using 1.5 Å root-mean-square-deviation, that is, RMSD, clustering during the docking process [49, 51, 52].

### 2.6. Active Site and Accessible Area Analysis

In order to have a better understanding of the interaction between the proteins DEPDC1B and Rac1, it becomes crucial to have a knowledge of the active site, the region in a protein that allows a protein to bind to a specific protein, of DEPDC1B (Figure 8). Additionally, active site of the template was predicted as well, in order to check for the preservation of structural similarity between the target and the template protein (Figure 4(c)). Protein Allosteric and Regulatory Sites (PARS), an online server (http://bioinf.uab.cat/cgi-bin/pars-cgi/pars.pl), was utilized for the purpose of identifying and characterizing the active site of the 3D model of the DEPDC1B protein and the template, GTPase-activation domain of RhoGAP (*Homo sapiens*) [34].

## 3. Results and Discussion

**Figure 7:**
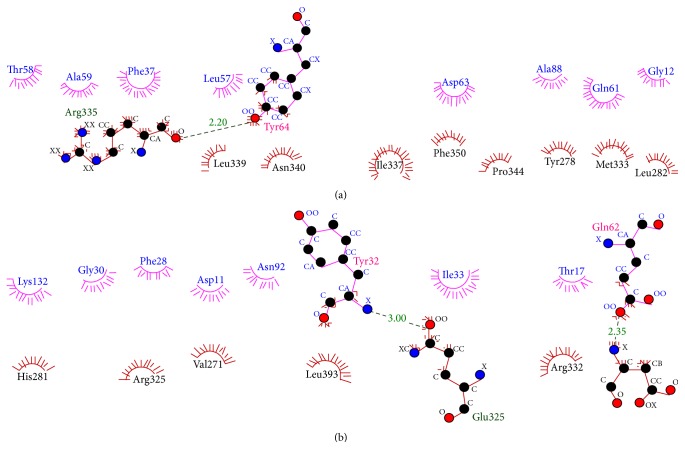
Interacting amino acid residues between DEPDC1B (red) and Rac1 (pink) visualized using LigPlot Plus [37]; the image has been illustrated as two halves (a) first half and (b) second half.

**Figure 8:**
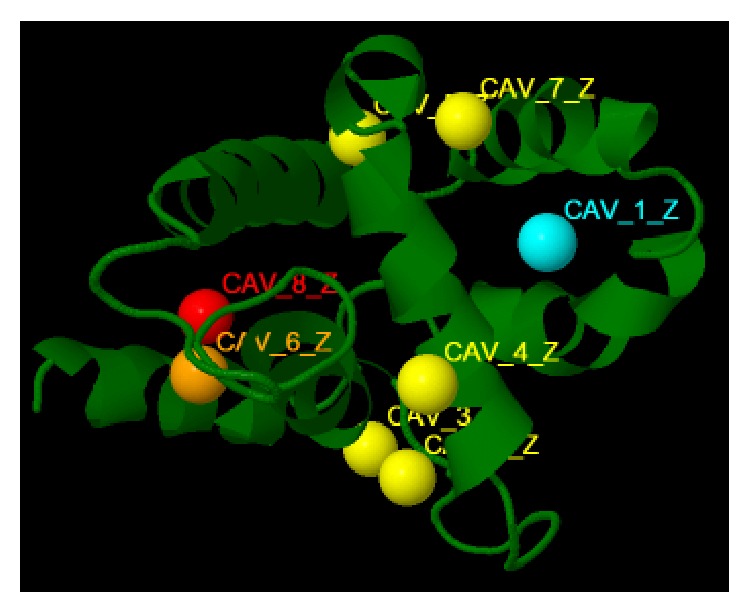
Potential allosteric or regulatory sites in the predicted model of DEPDC1B. Cyan colored pocket representing the structurally conserved sites and orange colored pockets representing the ones that affect the flexibility. Sites matching both criteria are marked red [34].

For the purpose of targeting a protein therapeutically it is central to understand the structural characteristics of the protein under study. However, the absence of the 3D model of the DEPDC1B protein makes it challenging to understand its structural and functional features. Therefore, to have a better understanding, 3D model of DEPDC1B is predicted in the study predicted using Swiss model and visualized using PyMol (Figure 2), wherein it was observed that the protein consists of six *α* helices. Pairwise structural alignment of the predicted model and the selected template indicated that the two structures were 20.97% identical and 33.06% similar, suggesting that the two structures are significantly similar (Figure 4(a)).  Figure 4 also illustrates the superimposition of template (grey in color) upon the modeled target protein, DEPDC1B (red in color) (Figure 4(b)). Interestingly, the comparison between Figure 4(b) obtained via superimposition of template upon DEPDC1B and Figure 4(c), depicting the possible active sites present in the template (PDB ID: 1RGP), indicated great similarity; that is, the most potential active site (red in color) in the template, CAV_4_Z (*p* value: 0.02 and structural conservation: 100, depicted in Table 1), was found to be structurally conserved in the protein DEPDC1B as implemented in the superimposed region. Table 1 depicts the *p* value and structural conservation of the other predicted regulatory or allosteric active site in the template. The model generated was then validated using PROCHECK, wherein 89.4% of the total amino acid residues were found to be present in the favorable region along with 9.7% of the amino acid residues in the generously allowed region and 0.9% that is only one residue (Thr288) being in the disallowed region which can be ignored. On the contrary, the Ramachandran plot generated using RAMPAGE indicated the presence of 115 amino acid residues that is approximately 94.3% in the favorable region, 7 amino acid residues (5.7%) in the allowed region, and no residue in the outlier region, indicating good quality of the protein structure (Figure 5). Also, the VERIFY 3D results showed that an average 3D-1D score of 45.97% of the total residues was greater than or equal to 0.2, indicating a good environmental profile of the model. The model had a good environmental profile as the *Z*-score was found to be −0.161. An ideal model must have the average *Z*-score close to “0”; therefore the predicted 3D model lies quite close to the ideal model. Further, DEPDC1B has been found to directly interact with [17] Rac1 protein, thereby mediating a downstream signaling process involved in oral carcinogenesis. A docking study was performed to understand the protein-protein interaction using the web server PatchDock/firedock (Figure 6), which showed a high docking score of −16.17 (global energy, i.e., binding energy of the solution), indicating a spontaneous interaction between the two and the interacting amino acid residues were found to be Asp270-Glu62, Glu328-Tyr32, Arg336-Tyr64, and so forth (Figure 7). The identification of the interacting or the functionally important amino acid residues will help the researchers to modify the function of the protein and thereby design/develop drugs that are highly efficient and specific for the target protein. Additionally, the understanding of protein-protein interaction (DEPDC1B-Rac1 protein interaction) will enable the researchers to further identify the pathways mediated via DEPDC1B, specifically associated with the disease, that is, oral cancer, and thereby predict its function, consequently, allowing the development of improved strategies to extirpate oral cancer. Also, active site and accessible surface area analysis was done using PARS, and the regions with the highest possibility of being regulatory or allosteric sites were identified and marked as shown in Figure 8.  Table 2 lists the possible allosteric or regulatory sites which have been ranked according to their potential of regulating the function of DEPDC1B protein, on the basis of flexibility *p* value (indicating the possibility with which a site may alter overall protein flexibility) and structural conservation, wherein the estimation of structural conservation within the protein done via PARS followed the LIGSITE^csc^ pocket identification method (an extension of LIGSITE, where csc stands for Connolly surface and conservation) [34]. Based on the analysis done CAV_1_Z site with a flexibility *p* value of 0.07 and structural conservation of 54.60 had the highest potential of being a possible regulatory site as it fulfilled the criteria based on both flexibility *p* value and structural conservation and CAV_7_Z had the lowest potential as it had structural conservation of only 18.20. Also, CAV_8_Z site shows relevant values for both criteria, that is, “0” flexibility *p* value and structural conservation of 63.60. The 3D structure and active site prediction shall help the researcher to design a pharmacophore in order to get a drug which interacts at the site and also the identification of the amino acid residues shall help the researcher to probably design mutants of the protein which shall be able to interact with higher or lower affinity which can help hugely in the downstream signaling study.

## 4. Conclusion

Oral cancer, the sixth most commonly occurring human cancer [5] with 90% of it being oral squamous cell carcinoma [53], often preceded by the precancerous lesions such as leukoplakia and erythroplakia [54], has high prevalence in Southeast Asian countries including India due to the consumption of tobacco as well as betel quid [55]. The major challenge is the prediction of the transition from premalignant form to carcinogenic form [56].

DEPDC1B is recently found to be overexpressed in oral cancer and is capable of mediating anchorage-independent growth of oral cancer cells and promoting cell growth and invasion by interacting with Rac1 protein [17], making it a potential proteomic based biomarker as well as a therapeutic target for the purpose of curing the disease. However, due to absence of 3D structure of the protein, the structural and functional characterization of the protein becomes challenging. In order to have a detailed understanding of its role in oral cancer, the present study focused on predicting the 3D model of the protein DEPDC1B by taking RhoGAP as the template structure with the help of Swiss model, which as per the results was close to the ideal model and had a good environmental profile. Apart from the structural characterization of a protein, it is of utmost importance to understand and identify the binding partners of the protein implicated in a disease, in this case interaction of DEPDC1B with other proteins, such as Rac1, as it allows the researchers to deduce/further predict and enhance our knowledge regarding the functional characteristics of the target protein. Rac1 which is known to play a crucial role in the onset of oral carcinogenesis, by regulating cell cycle, contact inhibition, cellular growth, and proliferation mediated via NFkB activation [23–26], indicates the possible pathway through which DEPDC1B initiates oral carcinogenesis. Further, the active site and accessible surface area analysis via PARS focused on the identification of the sites with the highest potential of being regulatory or allosteric sites, in order to therapeutically target the protein by specifically modifying or targeting the identified sites, wherein CAV_1_Z (Figure 8) was marked as the allosteric/regulatory site with the highest potential. The sites with moderate or less potential can also be further analyzed.

Therefore, by predicating the 3D model of DEPDC1B, the present study was able to identify the interacting amino acid residues of the protein DEPDC1B with the Rac1, through which DEPDC1B regulates the transition of a normal cell to a malignant one. Also, the identification of the possible allosteric or regulatory sites enhances our knowledge and thereby will play a crucial role in designing an appropriate drug that will have the potential of targeting the protein DEPDC1B and therefore curing the disease, that is, oral cancer.

## Figures and Tables

**Table 1:** Values indicating potential allosteric or regulatory sites of the template (PDB ID: 1RGP), estimated using the server PARS [34].

Rank	Site ID	Flexibility *p* value	Structural conservation
1	CAV_4_Z	0.02	100.00
2	CAV_5_Z	0.00	100.00
3	CAV_1_Z	0.03	36.40
4	CAV_2_Z	0.79	9.10
5	CAV_3_Z	0.70	0.00
6	CAV_7_Z	0.53	54.60
7	CAV_6_Z	0.83	9.10
8	CAV_8_Z	0.12	18.20

**Table 2:** Values indicating potential allosteric or regulatory sites of the target protein, DEPDC1B, estimated using the server PARS [34].

Rank	Site ID	Flexibility *p* value	Structural conservation
1	CAV_1_Z	0.07	54.60
2	CAV_8_Z	0.00	63.60
3	CAV_6_Z	0.03	18.20
4	CAV_2_Z	0.38	9.10
5	CAV_3_Z	0.64	18.20
6	CAV_4_Z	0.21	9.10
7	CAV_5_Z	0.07	9.10
8	CAV_7_Z	0.07	18.20
